# Development and Validation of a Recombinant VP2-Based Indirect ELISA for Canine Parvovirus

**DOI:** 10.3390/microorganisms14051161

**Published:** 2026-05-21

**Authors:** Bocheng Gao, Jiale Yi, Linna Gai, Jing Liu, Xuan Min, Ju Yao, Mingzhi Li, Jiarong Liu, Yule Chen, Su Wu, Yunzi Hu, Lingbao Kong

**Affiliations:** 1 Nanchang City Key Laboratory of Animal Virus and Genetic Engineering, College of Bioscience and Engineering, Institute of Pathogenic Microorganism, Jiangxi Agricultural University, Nanchang 330045, China; gaobocheng2005@163.com (B.G.); 13576936967@163.com (L.G.); 13627083137@163.com (X.M.);; 2 Key Laboratory of Agricultural Resource and Ecology in the Poyang Lake Basin of Jiangxi Province, School of Land Resources and Environment, Jiangxi Agricultural University, Nanchang 330045, China; yiyanmagic1@gmail.com

**Keywords:** canine parvovirus, VP2 protein, antibody detection, prokaryotic expression, indirect ELISA, bioinformatics

## Abstract

This study aimed to express the canine parvovirus (CPV) VP2 protein prokaryotically and develop an indirect ELISA for detecting CPV-specific antibodies in canine serum. The *VP2* gene from a laboratory-isolated CPV strain was amplified and cloned into the pET-28a vector. Following prokaryotic expression optimization, the recombinant protein was purified via Ni-NTA affinity chromatography and validated using Western blotting. An indirect ELISA was established utilizing the purified VP2 as the coating antigen, with optimal parameters determined by checkerboard titration. A 1773 bp VP2 fragment was amplified. Optimal expression of the 64.8 kDa recombinant VP2 was achieved with 2 mmol/L isopropyl β-D-thiogalactoside (IPTG) at 32 °C for 8 h. For the indirect ELISA, the optimal antigen coating concentration was 2 μg/mL, alongside primary (canine serum) and secondary antibody dilutions of 1:320 and 1:4000, respectively. The diagnostic cut-off optical density at 450 nm (OD_450_) threshold was established at ≥0.2066, and the analytical sensitivity reached a serum dilution of 1:5120. Compared with the hemagglutination inhibition (HI) assay using 192 clinical serum samples, the ELISA showed a diagnostic sensitivity of 85.94%, a diagnostic specificity of 88.28%, and an overall agreement rate of 87.50%. The mean intra-assay and inter-assay coefficients of variation were 4.39% and 3.02%, respectively. These findings indicate that the recombinant VP2-based indirect ELISA showed good analytical sensitivity, reproducibility, and diagnostic agreement with the HI assay for detecting CPV-specific antibodies in canine serum under the tested conditions, although broader cross-reactivity validation is still required.

## 1. Introduction

Canine parvovirus (CPV) is a highly contagious, non-enveloped, single-stranded DNA virus that primarily infects members of the family Canidae [1,2,3]. Classified within the family Parvoviridae, CPV was first recognized in the late 1970s and was formally confirmed in 1978 [2]. Within one to two years after its emergence, CPV spread rapidly worldwide and became a major pathogen affecting domestic and wild canids, causing substantial impacts on animal health and the canine industry. CPV is closely related to other carnivore parvoviruses, such as feline panleukopenia virus (FPV) and mink enteritis virus (MEV) [4,5]. CPV and FPV share high nucleotide sequence identity across much of their genomes, with only a few amino acid differences in the capsid proteins that are critical for host range determination [5].

Current evidence supports the hypothesis that CPV originated from FPV-like parvoviruses circulating in wild carnivores, followed by mutations that enabled adaptation to canine hosts [4]. CPV displays marked host adaptability and genetic plasticity and has diversified into the principal antigenic variants CPV-2a, CPV-2b, and CPV-2c [1]. Conventional vaccines prepared from CPV-2b generally provide cross-protection against these variants; however, vaccine efficacy may be affected by viral genetic variation, improper vaccination, or the use of expired or otherwise degraded vaccine preparations [5].

Susceptible hosts include domestic dogs and wild canids such as wolves and foxes [2,6,7]. Puppies, particularly those lacking maternally derived antibodies or vaccine-induced immunity, are especially vulnerable and may develop severe disease [8]. CPV is transmitted primarily via the fecal–oral route. Owing to its high environmental stability, the virus can remain infectious in contaminated feces, soil, or fomites for extended periods under typical environmental conditions [9]. Clinically, CPV infection mainly manifests as an enteric form characterized by severe vomiting and hemorrhagic diarrhea [10,11], or, less commonly, as a myocardial form that can lead to acute cardiac failure in neonatal animals [12]. Mortality in untreated severe cases is high and is generally associated with dehydration, electrolyte imbalance, and secondary infections [12].

CPV virions are small icosahedral particles, approximately 20–26 nm in diameter, and contain a genome of approximately 5000 nucleotides [2]. The viral capsid is mainly composed of VP2, a structural protein of approximately 584 amino acids with a molecular weight of about 64.8 kDa, which is closely related to the larger VP1 translation product [13,14]. In the assembled capsid, 60 viral proteins, predominantly VP2 with a minor proportion of VP1, form the icosahedral shell. VP2 is the major determinant of CPV host range, antigenicity, and virulence [15]. The VP2 gene evolves rapidly, with an estimated substitution rate on the order of 10^−4^ substitutions per site per year [5]. Amino acid changes at specific capsid residues have contributed to the emergence of CPV-2a, CPV-2b, and CPV-2c lineages, highlighting the importance of VP2 variation in CPV classification and antigenic characterization.

Rapid lateral-flow immunochromatographic assays, such as colloidal-gold antigen tests, are widely used for point-of-care detection of CPV antigens in clinical samples [16]. However, these assays may produce false results under certain conditions, and their performance can vary among products and sample types [17]. Serological assays that detect CPV-specific antibodies provide complementary information on host immune status, previous exposure, and vaccine-induced responses. Among these methods, enzyme-linked immunosorbent assays (ELISAs) offer advantages of high throughput, sensitivity, and specificity when properly optimized and validated [18,19]. Therefore, developing a reliable indirect ELISA based on the VP2 antigen is a feasible and valuable strategy for CPV serological surveillance and vaccine efficacy evaluation [20].

In the present study, we performed bioinformatic analyses of the VP2 gene from a CPV strain isolated in our laboratory, cloned the full-length VP2 open reading frame, and expressed the recombinant VP2 protein using a prokaryotic expression system. The recombinant protein was purified and verified by SDS-PAGE and Western blotting. Using the purified VP2 protein as the coating antigen, we established and optimized an indirect ELISA for detecting CPV-specific antibodies in canine serum samples. This study aimed to develop a reproducible, sensitive, and specific serological assay for CPV antibody detection, thereby providing technical support for CPV surveillance, immune evaluation, and related research applications.

## 2. Materials and Methods

### 2.1. Virus Strains, Bacterial Strains, Primary Materials, and Reagents

The CPV strain CPV_NC2025 was isolated from a diseased dog provided by the Guangwu Pet Hospital to the Key Laboratory of Animal Viruses and Genetic Engineering in Nanchang City. The pET-28a vector was maintained in our laboratory. Competent *E. coli* DH5α and BL21 cells were prepared in-house using a standard calcium chloride (CaCl_2_)-based method. Briefly, bacterial cultures were grown to mid-log phase (OD_600_ ≈ 0.35–0.40), chilled on ice, and treated with ice-cold CaCl_2_ solution to induce competence. The detailed preparation protocol is provided in the Supplementary Materials. Canine CPV-positive and negative sera were collected from the Pet Group of the College of Animal Science at Jiangxi Agricultural University. PrimeSTAR High Fidelity Enzyme, T4 DNA Ligase, XhoI, and BamHI restriction endonucleases were purchased from Bao Bioengineering Co., Ltd. (Dalian, China); DL5000/DL8000 DNA Marker was purchased from Beijing Kangrun Chengye Biotechnology Co., Ltd. (Beijing, China); Trans 180 kDa Protein Molecular Standard Marker and Goldview Nucleic Acid Gel Stain (10,000×) were purchased from Yisheng Biotechnology (Shanghai) Co., Ltd. (Shanghai, China); Plasmid extraction kits were purchased from Tiangen Biochemical Technology (Beijing) Co., Ltd. (Beijing, China); The DNA Purification and Recovery Kit was purchased from Sangon Biotech (Shanghai) Co., Ltd. (Shanghai, China); Ni-NTA Agarose was purchased from QIAGEN GmbH, Hilden, Germany; Kanamycin and Coomassie Brilliant Blue (R250) were purchased from Beijing Solarbio Technology Co., Ltd. (Beijing, China); Inclusion Body Wash Solution A (10 mL/L Triton X-100, 10 mmol/L Tris-HCl, 20 mmol/L NaCl, 0.2 mmol/L EDTA), Inclusion Body Wash Solution B (10 mmol/L Tris-HCl, 20 mmol/L NaCl, 0.2 mmol/L EDTA, 1 mmol/L β-Mercaptoethanol, 0.4 mol/L Urea), Inclusion Body Wash Solution C (10 mmol/L Tris-HCl); Purification Buffer A: 20 mM Tris-HCl, 500 mM NaCl, 8 M Urea, 1 mM β-Mercaptoethanol; Purification Buffer B: 20 mM Tris-HCl, 8 M Urea, 500 mM NaCl, 500 mM Imidazole; His-tag monoclonal antibody (mouse source) and horseradish peroxidase (HRP)-labeled goat anti-mouse antibody were purchased from Wuhan Sanying Biotechnology Co., Ltd. (Wuhan, China). HRP-rabbit anti-dog IgG (H + L) was purchased from Suzhou BioLong Technology Co., Ltd. (Suzhou, China). TMB color development solution was purchased from Shanghai Biyuntian Biotechnology Co., Ltd. (Shanghai, China).

### 2.2. Bioinformatics Analysis

#### 2.2.1. Physicochemical Properties and Phylogenetic Analysis

The amino acid sequence of the VP2 protein from the CPV_NC2025 isolate was compared with representative VP2 sequences from CPV and closely related parvoviruses. These reference sequences were retrieved from the NCBI GenBank database. To ensure data transparency and traceability, detailed epidemiological information for these sequences—including GenBank accession numbers, host species, geographical origin (country/region), and collection year—is provided in Supplementary Table S1.

The physicochemical properties of CPV_NC2025 VP2 and representative parvoviral proteins, including amino acid length, molecular weight, theoretical isoelectric point, and hydrophobicity, were analyzed using the ExPASy web program (https://www.expasy.org). For phylogenetic analysis, multiple sequence alignment of the VP2 amino acid sequences was performed using MUSCLE. The best-fit amino acid substitution model was selected using ModelFinder implemented in IQ-TREE according to the Bayesian information criterion (BIC). The maximum-likelihood (ML) phylogenetic tree was reconstructed using IQ-TREE under the VT+F+G4 model. Branch support was assessed using 1000 ultrafast bootstrap replicates and 1000 SH-aLRT replicates. To improve the transparency of the phylogenetic analysis, the branch labels in the tree were annotated with their respective accession numbers, host, country, and year of collection.

#### 2.2.2. Antigenicity and Structure Prediction

The IEDB online analysis platform (https://www.iedb.org/) was used to predict the B-cell antigen epitopes of the VP2 protein of the CPV-NC2025 isolate in order to evaluate its possible immunogenic regions. To determine the proportions of structural elements such as α-helices, β-sheets, extended regions, and random coils, the VP2 amino acid sequence was then sent to the SOPMA website (https://npsa.lyon.inserm.fr/) for protein secondary structure analysis. In order to create a three-dimensional structural model of the VP2 protein and provide a structural basis for examining its antigenic determinants and spatial conformation, the same sequence was also submitted to the SWISS-MODEL platform (https://swissmodel.expasy.org/) for protein tertiary structure prediction.

### 2.3. Gene Cloning and Expression

#### 2.3.1. Primer Design and PCR

The primers used for amplification of the CPV VP2 gene were designed using SnapGene 6.0.2 software based on the VP2 gene sequence of the laboratory-isolated strain CPV_NC2025. The forward primer CPV-VP2-F1 was 5′-CGCGGATCCCCAATGAGTGATGGA-3′ and contained a BamHI restriction site. The reverse primer CPV-VP2-R1 was 5′-CCGCTCGAGATATAATTTTCTAGGTGCTAGTTGA-3′ and contained an XhoI restriction site. The expected amplification fragment size was 1773 bp. The primers were provided by Shanghai Sangon Biotech Co., Ltd. PCR amplification was performed using the VP2 gene sequence from CPV_NC2025 as the template.

#### 2.3.2. Construction of Prokaryotic Expression Vectors

The VP2 target fragment was amplified using PrimeSTAR High Fidelity DNA polymerase. The PCR products were purified using the SanPrep Column PCR Product Purification Kit. The purified VP2 fragment and the pET-28a empty vector were double-digested with BamHI and XhoI. The digested target fragment was then ligated into the linearized pET-28a vector using T4 DNA ligase. The ligation products were transformed into competent *E. coli* DH5α cells, and the transformed cells were plated on LB agar medium containing 50 μg/mL kanamycin and incubated at 37 °C overnight. Positive colonies were selected and verified by colony PCR. The PCR-positive colonies were then transferred into liquid LB medium containing 50 μg/mL kanamycin for expansion culture. Recombinant plasmids were extracted using a plasmid mini-prep kit and further verified by double-enzyme digestion. The verified recombinant plasmids were subsequently sent to Shanghai Sangon Biotech Co., Ltd. for sequencing. The obtained sequencing results were compared with the CPV VP2 nucleotide sequence in GenBank. The successfully constructed recombinant expression plasmid was named pET-28a-VP2 and stored at −20 °C until further use.

#### 2.3.3. Optimization of Prokaryotic Expression Conditions

The recombinant plasmid pET-28a-VP2 was transformed into competent *E. coli* BL21 cells. The transformed cells were plated on LB agar medium containing 50 μg/mL kanamycin and incubated at 37 °C overnight. Positive colonies were verified by colony PCR and then inoculated into 5 mL of LB medium containing 50 μg/mL kanamycin, followed by incubation for 12 h to prepare the seed culture.

The seed culture was transferred into fresh LB medium containing 50 μg/mL kanamycin at a ratio of 1:100 and incubated at 37 °C with shaking at 220 rpm until the OD_600_ reached 0.4–0.6. Protein expression was induced by adding isopropyl-β-D-thiogalactoside (IPTG). To optimize the expression conditions, single-factor experiments were performed to evaluate the effects of IPTG concentration, induction temperature, and induction time. The IPTG concentrations tested were 0.2, 0.5, 0.8, 1.0, 1.5, and 2.0 mmol/L. The induction temperatures tested were 16, 24, 32, 37, and 42 °C. The induction times tested were 2, 4, 6, 8, 10, and 12 h.

After induction, 1 mL of bacterial culture was collected and centrifuged at 12,000 rpm for 5 min. The supernatant was discarded, and the bacterial pellet was resuspended in 160 μL of phosphate-buffered saline (PBS). The suspension was mixed with 40 μL of 5× protein loading buffer and heated in a metal bath for 20 min. The protein samples were then centrifuged at 12,000 rpm for 1 min, and the supernatants were analyzed by SDS-PAGE using a 10% gel to determine the optimal expression conditions.

Under the optimized induction conditions, VP2 protein was expressed in 500 mL of LB medium. The induced bacterial culture was collected by centrifugation at 12,000 rpm for 5 min, and the bacterial pellet was washed twice with PBS. The cells were resuspended in 20 mL of PBS and disrupted by sonication at 250 W using cycles of 15 s on and 45 s off for a total sonication time of 25 min. The lysate was centrifuged at 12,000 rpm at 4 °C for 10 min, and the supernatant and pellet fractions were separately collected. The pellet fraction was resuspended, and the expression pattern of VP2 protein was analyzed by SDS-PAGE using a 10% gel.

#### 2.3.4. Purification of VP2 Protein

After VP2 protein expression in inclusion bodies was confirmed, the inclusion bodies were washed twice with inclusion body wash solution A, followed by washing with inclusion body wash solutions B and C. The washed inclusion bodies were then dissolved overnight in purification buffer A. After centrifugation at 20,000 rpm for 30 min, the supernatant was collected, filtered through a 0.45 μm membrane, and reserved for purification. A Ni-chelating resin column was prepared and equilibrated with purification buffer A after natural settling. The protein sample was loaded onto the column, and the loading process was repeated to improve protein binding. Contaminating proteins were eluted with 30 mL of purification buffer A containing 40 mmol/L imidazole. The VP2 protein was then eluted with 10 mL of purification buffer A containing 80 mmol/L imidazole. The eluate was collected, mixed with protein loading buffer, and analyzed by SDS-PAGE.

#### 2.3.5. Western Blot Analysis of VP2 Protein

After SDS-PAGE electrophoresis, protein samples from the pET-28a empty vector control and the prokaryotically expressed VP2 protein were transferred onto a PVDF membrane using a transfer apparatus. The membrane was blocked with blocking solution for 2 h and washed three times with TBST. It was then incubated with mouse anti-His tag monoclonal antibody at a dilution of 1:5000 overnight at 4 °C. After three washes with TBST, the membrane was incubated with HRP-conjugated goat anti-mouse IgG at a dilution of 1:5000 for 2 h at 37 °C. After another three washes, the membrane was developed using a chemiluminescent substrate, and the immunoreactive bands were visualized.

### 2.4. Development of an Indirect ELISA Assay Method

#### 2.4.1. Determination of Optimal Antigen Coating Concentration and Serum Dilution

The optimal antigen coating concentration and primary antibody serum dilution were determined using a checkerboard titration method. The purified VP2 protein was diluted with coating buffer to final concentrations of 12, 10, 8, 4, 2, and 1 μg/mL. A total of 100 μL of each protein dilution was added to each microplate well, with two replicate wells prepared for each concentration. CPV-positive and CPV-negative canine sera were serially diluted from 1:20 to 1:40,960, and 50 μL of each serum dilution was added to the corresponding wells. HRP-conjugated rabbit anti-dog IgG was diluted to 1:4000, and 50 μL was added to each well. The assay was performed according to the standard ELISA procedure. The OD_450_ values of CPV-positive and CPV-negative sera were recorded as P and N values, respectively. The P/N ratio was calculated, and the combination with the highest P/N value was selected as the optimal antigen coating concentration and serum dilution.

#### 2.4.2. Optimal Dilution Ratio for Secondary Antibody

The optimized antigen concentration was added to microplate wells at 100 μL per well. CPV-positive and CPV-negative canine sera were added at the optimized dilution. HRP-conjugated rabbit anti-dog IgG was diluted with PBST at 1:1000, 1:2000, 1:4000, 1:8000, 1:16,000, and 1:32,000. Duplicate wells were prepared for each dilution, and 50 μL of each secondary antibody dilution was added to each well and incubated for 1 h. The subsequent ELISA procedure was performed as described above. The optimal secondary antibody dilution was determined based on the highest P/N value.

#### 2.4.3. Determination of the Cut-Off Value

A total of 64 CPV-negative canine serum samples were tested using the optimized indirect ELISA system, with two replicate wells prepared for each sample. The mean OD_450_ value and standard deviation ($\bar{SD}$) of the negative serum samples were calculated. The cut-off value was determined using the formula: cut-off = mean OD_450_ + 3 $\times \;\bar{SD}$. Samples with OD_450_ values equal to or greater than the cut-off value were considered positive, whereas samples with OD_450_ values below the cut-off value were considered negative.

### 2.5. Evaluation of the Indirect ELISA Assay

#### 2.5.1. Preliminary Analytical Specificity Testing

Six CPV-positive canine serum samples and canine distemper virus (CDV)-positive canine serum samples were tested using the optimized indirect ELISA. CPV-negative canine serum was used as the negative control. The OD_450_ values of CPV-positive and CDV-positive sera were measured and compared with the established cut-off value to preliminarily evaluate the analytical specificity of the assay.

#### 2.5.2. Analytical Sensitivity Testing

CPV-positive canine serum samples were serially diluted two-fold from 1:20 to 1:40,960, resulting in a total of twelve dilution levels. Each dilution was tested in triplicate using the established indirect ELISA. The OD_450_ value of each dilution was compared with the established cut-off value. The highest serum dilution with an OD_450_ value equal to or greater than the cut-off value was defined as the analytical sensitivity of the assay.

#### 2.5.3. Repeatability Testing

Eight CPV-positive canine serum samples with different antibody titers were tested using the established indirect ELISA. For intra-day repeatability, each serum sample was tested in triplicate under the same experimental conditions on the same day. For inter-day repeatability, the same serum samples were tested on different days using the same protocol. The OD_450_ values were recorded, and the coefficient of variation (CV) was calculated to evaluate the repeatability of the assay.

#### 2.5.4. Evaluation of Diagnostic Performance in Clinical Samples

To evaluate the diagnostic performance of the established indirect ELISA for CPV-specific antibody detection, 192 canine serum samples were collected and evaluated in a blinded manner. The hemagglutination inhibition (HI) assay, recommended by the World Organisation for Animal Health (WOAH), was used as the reference method. Receiver operating characteristic (ROC) curves were generated to assess the diagnostic performance of the indirect ELISA. The area under the ROC curve (AUC) and its corresponding 95% confidence interval (CI) were calculated using the ROC analysis module in GraphPad Prism software version 10.1.2 (GraphPad Software, San Diego, CA, USA). The optimal cut-off value was determined by maximizing the Youden index. Based on this cut-off value, the indirect ELISA results were dichotomized and cross-tabulated against the HI assay results to construct a 2 × 2 contingency table. Diagnostic sensitivity (DSe), diagnostic specificity (DSp), and the overall agreement rate were then calculated. Cohen’s Kappa coefficient was computed using SPSS 28.0.1 software to evaluate agreement between the indirect ELISA and the HI assay, with a Kappa value > 0.6 indicating substantial agreement.

## 3. Results

### 3.1. Physicochemical Properties and Phylogenetic Analysis of CPV_NC2025 VP2

**Figure 1:**
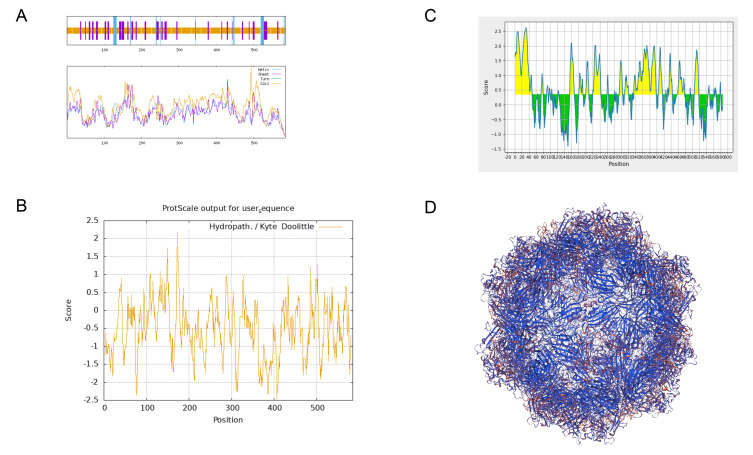
Bioinformatics analysis of the VP2 protein. (**A**) Prediction of the VP2 secondary structure. (**B**) Prediction of the VP2 hydropathy profile. (**C**) Prediction of VP2 B-cell antigenic epitopes. (**D**) Homology-modeled three-dimensional structure of the VP2 protein.

The physicochemical properties of CPV_NC2025 VP2 and representative parvoviral proteins were analyzed using ExPASy, and the detailed results are provided in Supplementary Table S2. CPV_NC2025 VP2 consisted of 585 amino acids, with a predicted molecular weight of 64.75 kDa, a theoretical isoelectric point of 5.53, a GRAVY value of −0.512, an instability index of 29.88, and an aliphatic index of 64.82. These results suggested that CPV_NC2025 VP2 is a hydrophilic and structurally stable protein, supporting its suitability for recombinant expression and antigen preparation (Figure 1B).

**Figure 2:**
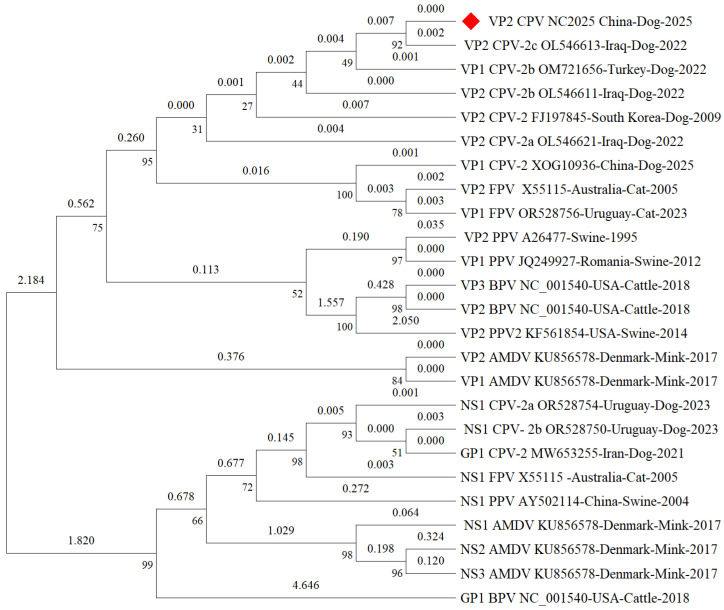
Maximum-likelihood phylogenetic tree based on amino acid sequences of CPV_NC2025 VP2 and representative VP2 proteins from CPV and closely related parvoviruses. The tree was reconstructed using IQ-TREE under the VT+F+G4 amino acid substitution model. Branch support values were calculated using 1000 ultrafast bootstrap replicates and 1000 SH-aLRT replicates. Sequence labels include accession number, host, country, and year of collection. The strain identified in this study is indicated by a red diamond. Numbers shown at the nodes represent bootstrap support values (%). The branch lengths correspond to genetic distances inferred from sequence divergence.

Phylogenetic analysis was performed using the maximum-likelihood method under the VT+F+G4 amino acid substitution model. The ML tree showed that CPV_NC2025 VP2 clustered within the CPV-2 lineage and was closely related to CPV-2c-like reference sequences (Figure 2). These results provided supporting information for the selection of CPV_NC2025 VP2 as the recombinant antigen for subsequent indirect ELISA development. However, because the analysis was based on a limited set of representative VP2 sequences, it was not intended to draw conclusions regarding antigenic drift, host adaptation, immune evasion, or the emergence of a new CPV lineage.

### 3.2. CPV-VP2 Antigenicity and Structural Prediction

The secondary structure of CPV_NC2025 VP2 was predicted using SOPMA (Figure 1A). And the detailed comparative results are shown in Supplementary Table S3. CPV_NC2025 VP2 was mainly composed of random coils and extended strands, accounting for 76.92% and 17.95%, respectively, whereas α-helices accounted for 5.13%. This structural distribution indicates that VP2 contains abundant flexible regions that may contribute to epitope exposure. B-cell epitope prediction further identified several potential antigenic regions on the VP2 protein surface, supporting its use as a coating antigen for the development of an indirect ELISA (Figure 1C). As seen in Figure 1D, the VP2 protein tertiary structure model created using homology modeling has a typical compact globular topology.

### 3.3. Amplification of the VP2 Gene

**Figure 3:**
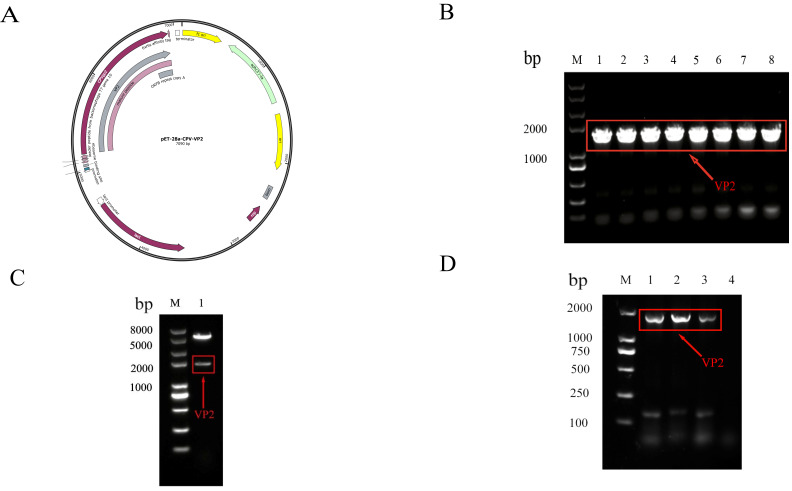
Construction and identification of the recombinant plasmid pET-28a-VP2. (**A**) Map of the recombinant plasmid pET-28a-VP2. (**B**) Identification by colony PCR. (**C**) Identification by double-enzyme digestion. (**D**) PCR amplification of the CPV VP2 gene. Red arrows and boxes indicate the target bands of the VP2 gene.

PCR amplification of the CPV_NC2025 VP2 gene produced a specific fragment of approximately 1773 bp, as confirmed by 1.0% agarose gel electrophoresis (Figure 3D).

### 3.4. Construction and Identification of the Recombinant Expression Vector

The recombinant plasmid pET-28a-VP2 was constructed as shown in Figure 3A. Colony PCR produced a specific band of approximately 1773 bp (Figure 3B), and double-enzyme digestion generated fragments consistent with the expected sizes (Figure 3C). These results confirmed the successful construction of the recombinant expression plasmid pET-28a-VP2.

### 3.5. Optimization of Recombinant VP2 Expression Conditions

The pET-28a-VP2 plasmid was transformed into competent *E. coli* BL21 cells, and recombinant VP2 expression was induced with IPTG. SDS-PAGE analysis showed a distinct band at approximately 64.8 kDa, consistent with the predicted molecular weight of the VP2 protein.

**Figure 4:**
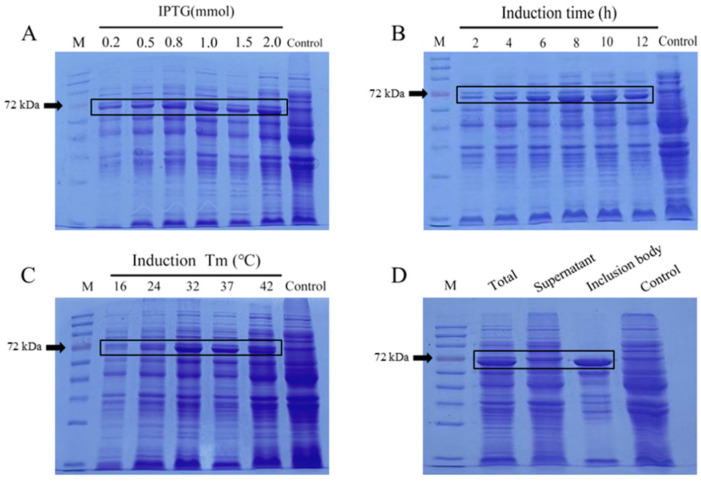
Optimization of prokaryotic expression conditions for VP2 protein. (**A**) Expression of VP2 under different IPTG concentrations. (**B**) Expression of VP2 at different induction temperatures. (**C**) Expression of VP2 at different induction times. (**D**) Solubility analysis of VP2 in bacterial cells.

To optimize recombinant VP2 expression, single-factor experiments were performed using different IPTG concentrations, induction temperatures, and induction times. Among the IPTG concentrations tested, the strongest VP2 expression was observed at 2.0 mmol/L IPTG (Figure 4A). Among the induction temperatures tested, 32 °C resulted in the highest expression level (Figure 4B). Among the induction times tested, VP2 expression reached its highest level after 8 h of induction (Figure 4C).

Therefore, the optimal prokaryotic expression conditions for recombinant VP2 were determined to be induction with 2.0 mmol/L IPTG at 32 °C for 8 h. Under these optimized conditions, VP2 was mainly detected in the inclusion-body fraction after sonication and centrifugation (Figure 4D).

### 3.6. Protein Purification for VP2

**Figure 5:**
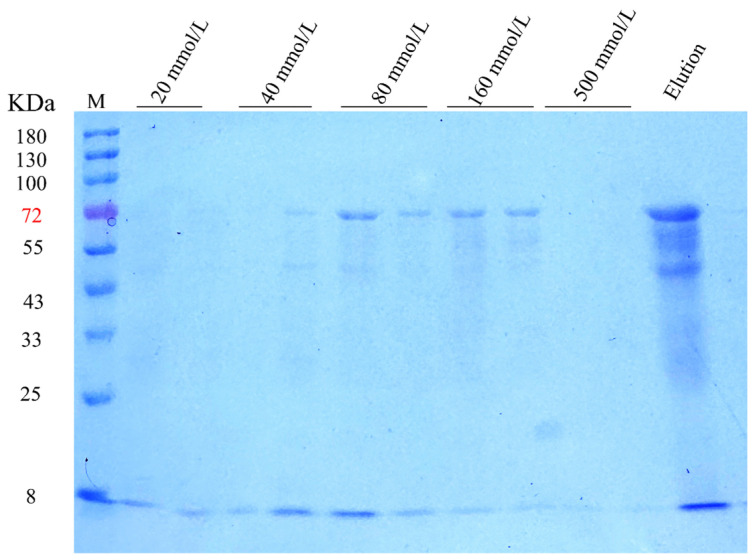
Separation and purification of the VP2 protein. The red‑labeled 72 kDa is the nearest molecular weight marker band to the theoretical 64.8 kDa of VP2 protein, assisting in locating the purified VP2 protein bands.

Following Ni-column chromatography purification of the VP2 protein, SDS-PAGE electrophoresis validation (Figure 5) identified a predominant band at approximately 64.8 kDa, which aligns with the theoretical molecular weight of the VP2 protein. Although a few minor bands of lower molecular weight were also observed—likely representing partially degraded fragments of the recombinant protein generated during the prokaryotic expression and inclusion-body purification processes—the target VP2 band remained the most prominent. Non-specific co-eluted proteins steadily declined among fractions eluted at varying imidazole concentrations, whereas the target band grew stronger. The 80 mmol/L imidazole eluate had the strongest target protein band. Quantitative analysis of the final purified product revealed a VP2 protein concentration of 0.8113 μg/μL. Given the total elution volume of 10 mL, the overall protein yield was calculated to be 8.113 mg. This yield was sufficient for downstream ELISA development and supports the feasibility of the optimized prokaryotic expression and purification strategy for preparing recombinant VP2 antigen.

### 3.7. Immunoblotting Identification of Recombinant Proteins

**Figure 6:**
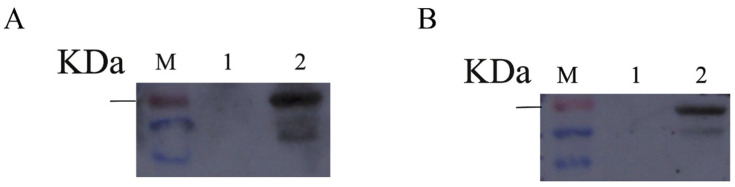
Immunoblot (Western blot) analysis of the CPV-VP2 protein. (**A**) Detection of VP2-specific expression using an anti-His tag antibody. (**B**) Detection of VP2 immunoreactivity using CPV-positive canine serum. M, protein marker; 1, uninduced sample; 2, induced sample.

Western blot analysis showed that a protein band of approximately 64.8 kDa was detected using the mouse anti-His tag monoclonal antibody, corresponding to the expected size of recombinant VP2 (Figure 6A). To further evaluate the immunoreactivity of the recombinant protein, Western blotting was also performed using CPV-positive canine serum. The expected 64.8 kDa band was recognized by CPV-positive canine serum (Figure 6B), indicating that the recombinant VP2 protein retained antigenic reactivity. However, additional weak bands were also observed, suggesting the presence of residual co-purified proteins, partially degraded VP2 fragments, or non-specific recognition by polyclonal antibodies in canine serum. Therefore, although the recombinant VP2 protein showed clear immunoreactivity, further purification and optimization may help reduce non-specific background.

### 3.8. Establishment of the Indirect ELISA Assay

**Figure 7:**
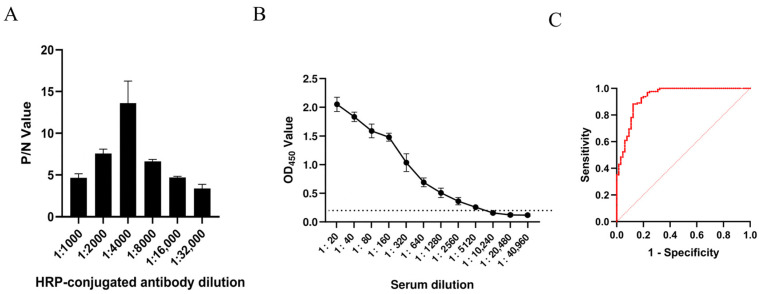
Establishment and clinical evaluation of the indirect ELISA detection system. (**A**) Optimization of the dilution factor for the HRP-conjugated antibody. (**B**) Determination of the analytical sensitivity of the indirect ELISA detection system; the horizontal dashed line indicates the established diagnostic cutoff point (OD_450_ = 0.2066). (**C**) ROC curve analysis of the established indirect ELISA using clinical serum samples. Error bars represent the $\bar{SD}$ of triplicate measurements.

Based on the ratio of OD_450_ values between positive and negative sera (P/N value), as well as practical experimental and production considerations for raw material conservation, the ideal protein coating concentration was found to be 2 μg/mL. The ideal dilution for both positive and negative sera was determined to be 1:320 (Table 1), while the enzyme-labeled secondary antibody required a dilution of 1:4000 (Figure 7A). The estimated mean was 0.1216, the $\bar{SD}$ was 0.0283, and the positive/negative threshold value was 0.2066 when testing canine negative serum using the established optimal detection system. Samples were deemed positive if their OD_450_ value was ≥0.2066; otherwise, they were deemed negative.

### 3.9. Evaluation of the VP2 Indirect ELISA Assay System

The OD_450_ value was 1.0805 for CPV-positive serum and 0.1505 for CDV-positive serum, indicating that the assay reacted with CPV-positive serum but not with CDV-positive serum under the tested conditions. These results suggested preliminary analytical specificity against CDV-positive serum. The analytical sensitivity of the assay reached a serum dilution of 1:5120, as determined by serial dilution testing (Figure 7B). The repeatability results of the indirect ELISA are shown in Table 2. For intra-day repeatability, the CV ranged from 2.48% to 6.30%. For inter-day repeatability, the CV ranged from 0.95% to 5.83%. All CV values were below 10%, indicating good repeatability and reproducibility of the established indirect ELISA.

To further evaluate the diagnostic performance of the established indirect ELISA for CPV-specific antibody detection, 192 canine serum samples were tested in parallel with the HI assay. Based on ROC curve analysis, the assay showed an AUC of 0.9344 (95% CI: 0.8957–0.9732, *p* < 0.0001). The optimal cut-off value, determined by maximizing the Youden index, was 0.2085, which was close to the preliminary cut-off value of 0.2066 calculated using the mean OD_450_ + 3 $\bar{SD}$ method. Using the ROC-derived cut-off value, the indirect ELISA correctly identified 55 of 64 HI-positive samples and 113 of 128 HI-negative samples (Table 3). Compared with the HI assay, the ELISA showed a diagnostic sensitivity of 85.94%, a diagnostic specificity of 88.28%, and an overall agreement rate of 87.50%. Cohen’s Kappa coefficient was 0.725 (*p* < 0.0001), indicating substantial agreement between the two methods.

## 4. Discussion

CPV is one of the most significant enteric pathogens affecting canids, posing a persistent threat to the global canine industry [2] and companion animal health due to its high transmissibility and mortality rates [21]. Although vaccination plays a pivotal role in disease control, the genetic variability of the virus may affect vaccine-induced protection under certain conditions, highlighting the need for serological tools to monitor CPV exposure and vaccine-induced antibody responses [15]. Moreover, the expanding scale of pet ownership exacerbates viral dissemination, underscoring the need for reliable serological tools to monitor CPV exposure and vaccine-induced antibody responses [6]. Among the existing CPV detection methods, the colloidal gold immunochromatographic assays offer rapidity and convenience, but their performance may vary among products, sample types, and testing conditions. In contrast, molecular techniques, such as PCR, require specialized equipment, limiting their utility for large-scale screening at the grassroots level. For instance, multiplex target PCR (MT-PCR) [22] enables the simultaneous detection of multiple targets [22,23]; however, it is hampered by the risk of cross-contamination during its two-step amplification process, which complicates procedures and reduces efficiency [24]. To address these gaps, the present study focused on the CPV capsid protein VP2, a key antigenic target, employing bioinformatics analysis, prokaryotic expression, purification, and reaction optimization to establish an indirect ELISA for detecting CPV antibodies in canine sera. This approach provides a technical tool for CPV-specific antibody monitoring, serological surveillance, and evaluation of vaccine-induced immune responses.

As the major capsid protein of CPV, VP2 contains important antigenic regions and has been widely used as a target antigen for CPV antibody detection and vaccine-related studies [25,26,27,28]. In the present study, bioinformatic and phylogenetic analyses were used only to support antigen selection and recombinant VP2 characterization. CPV_NC2025 VP2 clustered within the CPV-2 lineage and showed close relatedness to CPV-2c-like reference sequences, suggesting that it was a reasonable candidate antigen for developing a VP2-based indirect ELISA. However, because the present phylogenetic analysis included a limited number of reference sequences and the structural analysis was based on in silico prediction, these data are insufficient to support broader conclusions regarding antigenic drift, host adaptation, immune evasion, or the emergence of a new CPV branch [29]. Therefore, the significance of CPV_NC2025 VP2 in this study should be interpreted primarily in the context of recombinant antigen preparation and serological assay development [30].

In this study, we successfully amplified the full-length 1773 bp VP2 gene from the CPV_NC2025 strain and constructed the pET-28a-VP2 recombinant vector, which was verified through double restriction enzyme digestion and sequencing to confirm fidelity. Optimal expression conditions, determined via single-factor optimization, yielded high levels of VP2 production. SDS-PAGE and Western blot analyses confirmed a molecular weight of approximately 64.8 kDa for the recombinant protein. Following Ni-affinity chromatography purification, an enriched recombinant VP2 preparation was obtained, and the expected 64.8 kDa protein exhibited binding to anti-His monoclonal antibodies and CPV-positive canine sera, affirming the retention of VP2 immunoreactivity. These findings align with those of Park et al. [31], who utilized an *E. coli* prokaryotic system for CPV VP2 preparation, highlighting the advantages of this system in terms of simplicity, cost-effectiveness, and scalability for producing antigens suitable for serological assays.

In the Western blot analysis using CPV-positive canine serum, additional bands were observed besides the expected 64.8 kDa VP2 band. These bands may have resulted from residual bacterial proteins co-purified during Ni-NTA affinity purification, partial degradation or truncation of recombinant VP2 during inclusion-body solubilization and purification, or non-specific recognition by the polyclonal antibodies present in CPV-positive canine serum. Although the 64.8 kDa VP2 band was clearly detected and supported the antigenic reactivity of the recombinant protein, the presence of additional bands indicates that further optimization is needed to improve antigen purity and reduce background reactivity. Future improvements may include optimizing the imidazole washing and elution gradients, adding an additional purification step such as ion-exchange chromatography or size-exclusion chromatography, using protease inhibitors during protein extraction and purification, optimizing blocking and washing conditions in Western blotting, and adjusting the dilution of canine serum to reduce non-specific binding.

Using purified VP2 as the coating antigen, we developed an indirect ELISA protocol optimized via checkerboard titration, establishing optimal parameters: antigen coating concentration of 2 μg/mL, primary antibody dilution of 1:320, and secondary antibody dilution of 1:4000, with a cutoff value of 0.2066. Analytical specificity evaluation showed that the assay reacted with CPV-positive serum but not with CDV-positive serum under the tested conditions, suggesting preliminary analytical specificity. However, because only CDV-positive serum was included as a heterologous viral control, the cross-reactivity profile of this assay remains incompletely characterized. Further validation should include a broader panel of sera from dogs vaccinated against CPV, dogs exposed to related carnivore parvoviruses, and dogs infected or vaccinated with other common canine pathogens, such as canine coronavirus, canine adenovirus, canine parainfluenza virus, canine respiratory coronavirus, and canine influenza virus. The analytical sensitivity of the assay reached a serum dilution of 1:5120, supporting its ability to detect low-titer CPV-specific antibody samples under the optimized conditions. The intra- and inter-assay coefficients of variation were both below 10%, indicating good repeatability and reproducibility. Compared with the indirect ELISA for giant panda-derived CPV established by Li et al., both methods used VP2 as the core antigen and showed the same optimal coating concentration, supporting the suitability of VP2 as a coating antigen for CPV antibody detection [32]. These findings suggest that the recombinant VP2-based indirect ELISA may serve as a useful serological tool for CPV-specific antibody monitoring and vaccine-response evaluation, although broader cross-reactivity validation is still required before large-scale application.

In this study, 192 clinical sera were evaluated in parallel against the WOAH-recommended HI assay. The indirect ELISA showed an overall agreement rate of 87.50% with the HI assay, with a Cohen’s Kappa coefficient of 0.725, indicating substantial agreement between the two methods. The optimal ROC-derived cut-off value (0.2085) was close to the preliminary threshold (0.2066), supporting the robustness of the established cut-off under the tested conditions. Nevertheless, discrepant results were observed between the indirect ELISA and HI assay. These discrepancies may be partly related to differences in the principles of the two assays. The HI assay mainly reflects antibodies capable of inhibiting viral hemagglutination, whereas the indirect ELISA detects VP2-binding IgG antibodies. In addition, the recombinant VP2 antigen expressed in *E. coli* was obtained from inclusion bodies, and incomplete refolding or partial exposure of non-native epitopes may have affected antibody recognition in some samples. Differences in antibody titer, vaccination or exposure history, and sample background may also have contributed to the discordant results. Therefore, these discrepancies should not be attributed to a single factor, and further validation using larger and better-characterized serum panels is required. Several limitations should be acknowledged. First, the geographical, breed, and age diversity of the 192 clinical serum samples was limited. Second, although the established indirect ELISA showed no detectable reactivity with CDV-positive serum under the tested conditions, the analytical specificity of the assay has not yet been comprehensively evaluated. In the present study, sera from CPV-vaccinated dogs, dogs exposed to related carnivore parvoviruses, and dogs infected or vaccinated with other common canine pathogens were not included. Therefore, the cross-reactivity profile of the assay remains incompletely characterized and requires further validation using a broader panel of heterologous canine sera before large-scale application. Third, because this assay detects CPV-specific IgG antibodies, it cannot distinguish active infection from previous exposure, vaccination-induced antibodies, or maternally derived antibodies without additional clinical, epidemiological, or virological information. Therefore, the assay should be interpreted as a serological tool for CPV-specific antibody monitoring, CPV exposure assessment, and evaluation of vaccine-induced antibody responses, rather than as a standalone method for diagnosing active CPV infection.

Future studies should include broader serum panels with known vaccination, exposure, and infection histories to further evaluate the analytical specificity and diagnostic performance of the recombinant VP2-based indirect ELISA.

## 5. Conclusions

In conclusion, we developed a recombinant VP2-based indirect ELISA for detecting CPV-specific antibodies in canine serum. The assay showed acceptable analytical sensitivity, reproducibility, and diagnostic agreement with the HI assay under the tested conditions. Because the assay detects antibodies rather than viral antigen or nucleic acid, it should be used for serological surveillance, assessment of CPV exposure, and evaluation of vaccine-induced antibody responses, rather than as a standalone method for diagnosing active CPV infection. Further validation using broader serum panels with known vaccination, exposure, and infection histories is needed before large-scale application.

## Figures and Tables

**Table 1:** Determination of the optimal antigen coating concentration and serum dilution.

Coated Antigen (μg/mL)	OD_450_	Serum Dilution											
		1:20	1:40	1:80	1:160	1:320	1:640	1:1280	1:2560	1:5120	1:10,240	1:20,480	1:40,960
12	P	1.5192	1.5905	1.7837	1.501	1.1633	0.8227	0.6738	0.4568	0.226	0.1868	0.1379	0.1318
	N	0.0895	0.0979	0.1083	0.1079	0.0906	0.1121	0.083	0.0923	0.1277	0.1247	0.1236	0.1093
	P/N	16.9743	16.24617	16.46999	13.91103	12.83996	7.338983	8.118072	4.949079	1.769773	1.497995	1.115696	1.205855
10	P	1.6089	1.8391	1.7839	1.4627	1.1249	0.7797	0.6466	0.3772	0.2316	0.1846	0.1448	0.1566
	N	0.1088	0.102	0.1134	0.1105	0.1216	0.1134	0.128	0.1239	0.1252	0.1215	0.125	0.1274
	P/N	14.78768	18.03039	15.73104	13.2371	9.250822	6.875661	5.051563	3.044391	1.84984	1.519342	1.1584	1.229199
8	P	1.8546	1.7707	1.7794	1.5051	1.165	0.8702	0.578	0.4323	0.245	0.187	0.1507	0.168
	N	0.1035	0.1024	0.1023	0.1048	0.1083	0.1063	0.1118	0.11	0.1176	0.1129	0.1141	0.1327
	P/N	17.91884	17.29199	17.39394	14.36164	10.75716	8.186265	5.169946	3.93	2.083333	1.656333	1.320771	1.266014
4	P	1.7544	1.8243	1.7625	1.519	1.1954	0.8991	0.7148	0.4102	0.2458	0.1811	0.1561	0.1562
	N	0.1073	0.1079	0.1078	0.1113	0.1122	0.1095	0.1044	0.1184	0.1095	0.1149	0.1084	0.1017
	P/N	16.35042	16.90732	16.34972	13.6478	10.65419	8.210959	6.846743	3.464527	2.244749	1.576153	1.440037	1.53589
2	P	2.0773	1.8965	1.6256	1.342	1.0175	0.6819	0.5194	0.3454	0.2078	0.1516	0.1287	0.1102
	N	0.1389	0.0867	0.0876	0.0881	0.0861	0.092	0.0859	0.0889	0.0831	0.0843	0.0845	0.0878
	P/N	14.95536	21.87428	18.55708	15.23269	11.81765	7.411957	6.046566	3.885264	2.500602	1.798339	1.523077	1.255125
1	P	1.888	1.6458	1.3777	1.1213	0.8379	0.5387	0.3941	0.2665	0.1862	0.1464	0.1263	0.1142
	N	0.0925	0.0902	0.0896	0.0899	0.0892	0.0868	0.084	0.0879	0.0878	0.1011	0.0833	0.0879
	P/N	20.41081	18.24612	15.37612	12.47275	9.393498	6.206221	4.691667	3.031854	2.120729	1.448071	1.516206	1.299204

**Table 2:** Repeatability assay of the indirect ELISA system.

Sample No.	OD_450_ Values for Intra-Day Repeatability			OD_450_ Values for Inter-Day Repeatability		
	Mean	SD	CV	Mean	SD	CV
1	0.7632	0.0351	4.60%	0.7559	0.0104	1.38%
2	0.8805	0.0449	5.10%	0.8714	0.0128	1.47%
3	1.1220	0.0671	5.98%	1.0832	0.0549	5.07%
4	0.9231	0.0268	2.90%	0.9293	0.0088	0.95%
5	0.6915	0.0171	2.48%	0.7028	0.0159	2.26%
6	1.1706	0.0737	6.30%	1.2034	0.0463	3.85%
7	0.5758	0.0235	4.08%	0.6006	0.0350	5.83%
8	0.8029	0.0294	3.66%	0.8225	0.0277	3.37%

**Table 3:** Comparison of the indirect ELISA and HI test results for clinical canine serum samples.

	HI Positive	HI Negative	Total
ELISA Positive	55	15	70
ELISA Negative	9	113	122
Total	64	128	192

## Data Availability

The original contributions presented in this study are included in the article/Supplementary Materials. Further inquiries can be directed to the corresponding author.

## Supplementary Materials

The following supporting information can be downloaded at: https://www.mdpi.com/article/10.3390/microorganisms14051161/s1, Table S1: Amino acid and nucleotide sequences of CPV‑VP2 and related parvovirus reference strains; Table S2: Comparative physicochemical properties of the VP2 gene of the CPV isolate CPV_NC2025 and homologous parvoviral genes; Table S3: Comparative secondary structure features of the VP2 protein from the CPV isolate CPV_NC2025 and related viral genes.
