# Supplementary material for: Development and Validation of a Recombinant VP2-Based Indirect ELISA for Canine Parvovirus
Source: Microorganisms. 2026 May 21;14(5):1161. doi: 10.3390/microorganisms14051161 (PMC13210127; doi:10.3390/microorganisms14051161)
Supplement: Supplementary file 1 [file microorganisms-14-01161-s001.zip › Supplementary Materials.pdf]

Supplementary Information

Development and Validation of a Recombinant VP2-Based Indirect ELISA for Canine Parvovirus

This file includes :

A. Supplementary Notes:

E. coli DH5α and BL21 strains were cultured in Luria-Bertani (LB) medium at 37 °C with vigorous shaking until the optical density at 600nm (OD_{600}) reached 0.35–0.40. The bacterial cultures were chilled on ice for 10 min and subsequently harvested by centrifugation at 4,100 rpm for 10 min at 4 °C. The cell pellets were gently resuspended in 30 mL of ice-cold 0.1M $CaCl_2$-$MgCl_2$ solution (80mM $MgCl_2$, 20mM $CaCl_2$) per 50 mL of initial culture. Following a second centrifugation step under identical conditions, the pellets were resuspended in 2 mL of ice-cold 0.1M $CaCl_2$. The prepared competent cells were aliquoted and stored at -80°C prior to transformation.

B. Supplementary Tables:

Table S1. sequence

Refer to Excel Table S1 for sequence data

Table S2. Comparative physicochemical properties of the *VP2* gene of the CPV isolate CPV_NC2025 and homologous parvoviral genes.

Gene Name	Amino acids length	Molecular weight	Theoretical isoelectric point	GRAVY	Instability index	Aliphatic index
CPV_NC2025	585	64751.15	5.53	-0.512	29.88	64.82
VP2 CPV-2	584	64574.83	5.52	-0.506	27.63	64.61
VP2 CPV-2a	584	64599	5.61	-0.486	27.69	65.1
VP2 CPV-2b	584	64595.94	5.44	-0.492	28.31	65.27
VP2 CPV-2c	584	64638.04	5.53	-0.503	29.56	64.93
VP1 CPV-2	727	80372.98	7.67	-0.588	33.88	63.34
VP1 CPV-2b	727	80246.72	7.67	-0.591	33.11	63.34
NS1 CPV-2a	668	76763.29	5.9	-0.54	41.03	75.3
NS1 CPV-2b	668	76732.19	5.77	-0.541	41.97	74.72
GP1 CPV-2	668	76764.23	5.77	-0.54	41.44	75.3
VP2 FPV	584	64695.12	5.44	-0.499	28.27	65.43
VP1 FPV	727	80403.95	7.67	-0.599	32.95	62.67
NS1 FPV	668	76768.27	5.9	-0.534	42.05	75.3

VP2 PPV	579	64409.91	6.19	-0.583	35.53	67.25
VP1 PPV	729	80855.28	8.11	-0.688	40.9	61.88
NS1 PPV	662	75646.95	6.64	-0.559	42.9	72.95
VP2 AMDV	746	81594.77	5.33	-0.498	36.69	60.38
VP1 AMDV	690	78467.22	5.73	-0.78	35.74	62.88
VP3 BPV	536	60220.53	8.18	-0.56	33.73	72.37
VP2 BPV	673	75103.38	9.27	-0.614	32.61	70.15
VP2 PPV2	555	61665.14	6.28	-0.531	46.02	66.5
GP1 BPV	726	81189.08	5.12	-0.541	42.15	72.81
NS1 AMDV	641	72574.57	8.67	-0.712	37.97	73.48
NS2 AMDV	114	13551.26	6.88	-1.184	58.42	51.49
NS3 AMDV	92	10798.13	8.98	-1.316	75.07	47.83

Table S3. Comparative secondary structure features of the VP2 protein from the CPV isolate CPV_NC2025 and related viral genes.

Gene Name	Alpha helix	Beta turn	Extended strand	Bend region	Random coil
VP2 CPV_NC2025	5.13%	0	17.95%	0	76.92%
VP2 CPV-2	4.45%	0	15.75%	0	79.79%
VP2 CPV-2a	4.79%	0	16.44%	0	78.77%
VP2 CPV-2b	4.11%	0	16.95%	0	78.94%
VP2 CPV-2c	3.60%	0	17.98%	0	78.42%
VP1 CPV-2	10.45%	0	15.13%	0	74.42%
VP1 CPV-2b	9.90%	0	14.17%	0	75.93%
NS1 CPV-2a	34.88%	0	8.08%	0	57.04%
NS1 CPV-2b	37.13%	0	7.04%	0	55.84%
GP1 CPV-2	39.52%	0	7.78%	0	52.69%
VP2 FPV	4.62%	0	18.49%	0	76.88%
VP1 FPV	10.18%	0	12.65%	0	77.17%
NS1 FPV	41.17%	0	6.29%	0	52.54%
VP2 PPV	5.01%	0	20.03%	0	74.96%
VP1 PPV	10.15%	0	12.89%	0	76.95%
NS1 PPV	37.31%	0	10.12%	0	52.57%
VP2 AMDV	10.72%	0	13.00%	0	76.27%
VP1 AMDV	4.35%	0	17.25%	0	78.41%
VP3 BPV	5.22%	0	21.27%	0	73.51%
VP2 BPV	11.74%	0	12.78%	0	75.48%
VP2 PPV2	4.86%	0	20.54%	0	74.59%
GP1 BPV	32.64%	0	5.92%	0	61.43%
NS1 AMDV	38.22%	0	8.27%	0	53.51%
NS2 AMDV	54.39%	0	4.39%	0	41.23%
NS3 AMDV	33.70%	0	9.78%	0	56.52%
